# Assessing vaginal microbiome through Vaginal Microecology Evaluation System as a predictor for *in vitro* fertilization outcomes: a retrospective study

**DOI:** 10.3389/fendo.2024.1380187

**Published:** 2024-07-09

**Authors:** Quan Tian, Shengxi Jin, Guangmin Zhang, Yujie Liu, Jianxin Liu, Xiuming Tang, Yufeng Li, Jiane Liu, Yifei Liu, Zheng Wang

**Affiliations:** ^1^ Department of Genetics and Cell Biology, Basic Medical College, Qingdao University, Qingdao, Shandong, China; ^2^ Department of Reproductive Medicine, The Affiliated Hospital of Qingdao University, Qingdao, Shandong, China; ^3^ Division of Reproductive Endocrinology and Infertility, Department of Obstetrics and Gynecology, Pennsylvania State Hershey Medical Center, Hershey, PA, United States

**Keywords:** infertility, IVF, dysbiosis, pregnancy outcomes, vaginal microecology

## Abstract

**Objective:**

This study aims to evaluate the effectiveness of the Vaginal Microecology Evaluation System (VMES) in assessing the dynamics of the vaginal microbiome (VM) throughout the process of *in vitro* fertilization and embryo transfer (IVF-ET). Furthermore, it seeks to explore the potential correlation between distinct types of VM ecology and the success rate of IVF-ET.

**Methods:**

This study employed VMES to ascertain the composition of the VM. Data were collected from infertile women who underwent their initial IVF-ET treatment for tubal factor between January 2018 and December 2021. A retrospective analysis of pregnancy outcomes resulting from their fresh embryo transfer was conducted to determine the predictive significance of the vaginal microenvironment.

**Results:**

We demonstrate that VMES is able to predict IVF-ET outcomes in patients diagnosed with Bacterial Vaginosis (BV). Notably, a discernible shift in the VM was observed in a decent subset of patients following Controlled Ovarian Stimulation (COS), though this phenomenon was not universal across all participants. Specifically, there was a noteworthy increase in the proportion of patients exhibiting BV and uncharacterized dysbiosis subsequent to COS. Furthermore, our investigation revealed a significant correlation between VM and both the live birth rate and early miscarriage rate. Employing a multivariable logistic regression model, we identified that VM status pre-COS, VM status post-COS, patient age, and the number of embryos transferred emerged as independent predictors of the live birth rate.

**Conclusion:**

Our study suggests that, during IVF-ET treatment, the VMES can effectively detect changes in the VM, which are strongly correlated with the pregnancy outcome of IVF-ET procedures.

## Introduction

The challenges of spontaneous conception affect approximately 15% of couples worldwide conception (1). Assisted reproductive technology (ART) serves as a solution for infertility, predominantly with *in vitro* fertilization (IVF) being the widely practiced procedure. IVF, conducted through either conventional insemination or intracytoplasmic sperm injection (ICSI), followed by embryo transfer (ET), stands as a commonly employed ART method. Despite the incorporation of diverse ovarian stimulation protocols and innovative embryo selection techniques over the past decades, the live birth rate (LBR) in ART has seen limited improvement (2). While various euploid embryo selection methods, such as noninvasive and minimally invasive preimplantation genetic testing for aneuploidy (PGT-A), show promise in enhancing pregnancy rates, they often overlook the crucial role played by the vaginal and uterine environment in sustaining pregnancy. The endometrial receptivity analysis appears beneficial for a specific subset of patients in determining the optimal ET timing window. However, its universal application is hindered by its time and cost inefficiency in evaluating uterine receptivity. There is a pressing need for a simple and feasible method applicable to all infertility patients for predicting pregnancy success rates. Such a method could significantly alleviate the physical, emotional, and financial burdens on IVF patients by enabling the delay of ET if additional treatments are warranted. Therefore, new insights are essential to enhance ART outcomes, especially considering the escalating number of ART treatments performed in recent years (3).

The amalgamation of vaginal micro-organisms, their genetic information, and the surrounding environment is collectively referred to as the vaginal microbiome (VM) (4). This microbiome recently emerged as an important player in maintaining a normal physiological environment crucial for reproductive success (5). Various factors, including both endogenous and exogenous, as well as sexual hormones, influence the composition of vaginal micro-organisms (6). Among these factors, hormonal status, in particular, dictates the microflora residing on the vaginal epithelial mucosa and impacts susceptibility to infection (7). Controlled ovarian stimulation (COS) and IVF-ET for infertility treatment present a unique opportunity to examine the VM in a shifting hormonal milieu and explore potential correlations between the VM and IVF cycle outcomes. Several reports have utilized molecular methods, such as 16S ribosomal RNA (16S rRNA) gene sequencing, to investigate the impact of short-term estrogen changes on the VM after COS. Hyman et al. proposed that the VM undergoes a transition during therapy in some but not all patients (8), while Carosso et al. reported significant changes in the composition of vaginal and endometrial microbiomes (9). Over the past decade, numerous studies have identified associations between the presence of microorganisms and ART outcomes, including implantation rates (IR), pregnancy rates, ongoing pregnancy rates, and LBR (8, 10, 11). Low abundance of vaginal bacteria was shown to be associated with an increased risk of preterm delivery (12), and, interestingly, colonization with hydrogen peroxide-producing *L. crispatus* has been shown to significantly improve implantation rates IR and LBR, highlighting the critical role of microbiome in controlling outcomes of ART (13–19). Intriguingly, abnormal uterine microbiomes significantly impact ART outcomes, as demonstrated in studies using culture-based methods and metagenomic sequencing, such as 16S rRNA gene sequencing (17, 20, 21). However, the study used qPCR assays targeting only 6 vaginal microbial species. Whatever, culture-based technology, qPCR and 16S rRNA sequencing encounter biases originating from *in vitro* culture, and limitations in analyzing a small number of samples due to high costs and low sequencing throughput. The depth of sample analysis provided by these methods is often unsuitable for clinical applications, and daily practice. Therefore, there is an urgent need to develop a simple diagnostic tool to comprehensively understand VM dynamics during IVF and to predict patients at risk of adverse reproductive outcomes.

In 2016, a standard tool known as the Vaginal Microecology Evaluation System (VMES) was issued by the Committee of Infectious Disease Collaborative Group to assess VM in China (22). VMES primarily relies on morphological diagnostic techniques and additionally utilizes five functional indicators to depict lower genital tract infections and indicate microbial functional status (23). Morphological indicators include bacterial density, flora diversity, predominant microbiota, white blood cell count, pathogens, Nugent score for bacterial vaginosis (BV) (24), and aerobic vaginitis (AV) score for AV (25). In addition, five functional indicators involve hydrogen peroxide (H_2_O_2_), sialidase, leukocyte esterase, beta-glucuronidase, and coagulase (26, 27). As such, the VMES system significantly aids clinicians in diagnosing and managing a broad spectrum of vaginal infections. Importantly, this system has successfully unraveled the causal link between vaginal dysbiosis and cervical cancer, elucidating the pathway involving oncogenic human papillomavirus acquisition, persistence, and cervicovaginal dysplasia development (28, 29). Yet, whether the VMES-mediated VM testing can be applied broadly in other disease settings has not been firmly established.

Here, we performed a retrospective analysis using VMES data alongside pregnancy outcomes obtained from infertile women undergoing IVF treatment. We found that VMES can effectively detect changes in vaginal microecology during ART treatment and showed that vaginal microecology strongly correlates with IVF-ET pregnancy outcomes, implying the potential application of VMES in evaluating pre-embryo transfer microbiome status as a valuable, independent predictor of ART outcomes.

## Methods

### Subjects

This is a retrospective cohort study that was approved by the Ethical Committee of the Affiliated Hospital of Qingdao University, China (Ref: QYFYWZLL26140). This study was performed according to the ethical guidelines which are issued by the Ethics Committee of the China Society of Obstetrics and Gynecology. Informed consent was obtained from all individual participants included in the study. Clinical data of 440 patients at the IVF center of the Affiliated Hospital of Qingdao University in Qingdao from January 2018 to December 2021, were included in this study. Inclusion criteria were: (i) patients undergoing their first gonadotrophin-releasing hormone analogue (GnRH-a) prolonged protocols for IVF-ET due to tubal factors; (ii) patients aged between 20 and 42 years with a male partner; (iii) patients with BMI values between 18 and 28 kg/m^2^; (iv) patients with anti-müllerian hormone (AMH) levels greater than 1.1 ng/ml; (v) patients with a normal menstrual cycle ranges from 21 to 35 days; (vi) patients with no history of recurrent miscarriage. Patients were excluded from the study if they had: (i) complaints of vaginal itching, burning, and dysuria; (ii) systemic antibiotics treatment within 4 weeks before IVF or IVF-ICSI; (iii) hormonal medication within 3 months before IVF or IVF-ICSI; (iv) pre-treatment with a GnRH-a and Endometriosis American Fertility Score III/IV; (v) physical diseases including cardiovascular, respiratory, endocrine, chronic autoimmune, or blood system diseases.

### Controlled ovarian stimulation and IVF-ET

**Figure 1:**
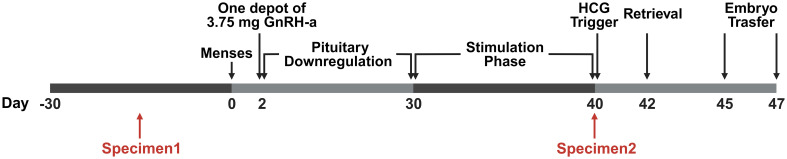
The timeline of the stimulus program and specimen acquisition. The study followed a Gonadotropin-Releasing Hormone agonist (GnRH-a) prolonged protocol within the ART procedures. On day 2 of the menstrual cycle, a 3.75 mg dose of GnRH-a was administered. Subsequently, recombinant Follicle-Stimulating Hormone (rFSH) was administered daily in doses ranging from 75 to 300 IU after confirming down-regulation through ultrasound scans and serum hormone level assessments. Upon reaching a mean diameter of 18 mm for the two leading follicles, a trigger for ovulation was induced using 6000–10,000 IU of Human Chorionic Gonadotropin (HCG). Transvaginal oocyte retrieval was performed 36–37 hours post-trigger. Fertilization was achieved through standard IVF or intracytoplasmic sperm injection (ICSI) methods. Embryo transfer took place on either day 3 or day 5 after fertilization. Specimen 1 was obtained 30 days ahead of pituitary downregulation, and Specimen 2 was obtained on the trigger day.

All patients underwent ovarian stimulation with the GnRH-a prolonged protocol (**Figure 1**). In brief, GnRH-a (3.75 mg leuprolide acetate; Shanghai Livzon Pharmaceutical Co., Ltd.) was given on the second day of the menstrual cycle. Subsequently, recombinant follicle-stimulating hormone (rFSH) was injected daily. Upon the attainment of a mean diameter of 18 mm in two leading follicles, human chorionic gonadotropin (hCG) (Livzon Pharmaceutical Group Inc., China) was administered. Oocyte retrieval took place 36–37 hours after the administration of the trigger shot. Fertilization was achieved using either standard IVF or ICSI. Embryo transfer was conducted on the third or fifth-day post-fertilization. Luteal phase support commenced on the day of oocyte retrieval and continued until the first serum hCG testing. Pregnancy was assessed by measuring serum hCG on the 14th day post-embryo transfer, and confirmation was later obtained through transvaginal ultrasound at 3 weeks. Luteal phase support was sustained until 10 weeks of gestation if gestational sac and fetal heart activity were observed. During GnRH-a prolonged protocol, serum E2 levels were recorded at baseline, on the first day of GnRH-a administration, and on the day of hCG trigger administration.

### Definition of clinical outcomes

In this study, the clinical outcomes were categorized as follows: (i) Biochemical pregnancy: defined with serum β-hCG ≥ 10 mIU/ml on the 14th day after ET; (ii) Clinical pregnancy: defined through the detection of the gestational sac by transvaginal ultrasound on the 35th day after ET; (iii) Early miscarriage: defined as s spontaneous abortion within 12 weeks of pregnancy; (iv) Live birth: defined as the delivery of any viable infants after 24 weeks; Among these outcomes, clinical pregnancy rate (CPR) and LBR were considered primary outcomes criteria, while IR, biochemical pregnancy rate (BPR) and early miscarriage rate (EMR) served as secondary outcomes. The specific calculation formulas used in the study were: (i) LBR per fresh transplantation cycle = number of patients with live birth/number of fresh embryo transfer cycles × 100%; (ii) CPR = number of clinical pregnancies/number of fresh embryo transfer cycles × 100%; (iii) IR = number of gestational sac/number of transferred embryos × 100%; (iv) BPR = number of biochemical pregnancies/number of fresh embryo transfer cycles × 100%; (v) EMR = number of early miscarriages/number of clinical pregnancies × 100%.

### Vaginal secretion collection

Vaginal microbiota specimens were systematically collected at two specific time points during this study: before the commencement of COS (pre-COS) and subsequent to the conclusion of COS (post-COS). Specifically, specimen 1 was acquired before pituitary downregulation, and specimen 2 was obtained on the day of hCG administration, as illustrated in **Figure 1**. The sampling protocol adhered to precise criteria: (i) the absence of menstruation for a minimum of 3 days; (ii) a 24-hour interval without vaginal intercourse, baths, or vaginal irrigation preceding the sampling; (iii) no administration of vaginal medications within 48 hours prior to sampling.

Vaginal discharge was meticulously collected from the posterior vault and upper third of the vagina, employing two long sterile cotton swabs. One of the cotton swabs was carefully placed in a test tube containing a small quantity of 0.9% sodium chloride solution, while the other cotton swab was deposited in a clean test tube. Both test tubes underwent immediate examination within an hour of collection.

### VMES

The VMES primarily comprises morphological and functional microecological indicators. Morphological examinations encompass bacterial density, flora diversity, dominant bacterial flora, trichomonads, spores, budding yeast, or hyphal forms for Vulvovaginal Candidiasis (VVC), Nugent scoring for BV, and AV scoring for AV (22, 30). Functional indicators encompass five preformed enzymes: H_2_O_2_, leukocyte esterase activity, sialidase activity, β-glucuronidase, and coagulase activity.

Patients diagnosed with vaginal infectious diseases received appropriate treatment. VVC or BV patients underwent treatment through vaginal administration of Metronidazole, Clotrimazole, and Chlorhexidine Acetate Effervescent Tablets for a duration of 3–5 days.

#### Morphological examination

The procedure involved smearing the dry cotton swab onto two clean slides. One slide underwent drying, fixation, and Gram staining, while the other was subjected to a drop of 0.9% sodium chloride and covered with a glass coverslip. Bacterial density, flora diversity, dominant bacterial flora, and Nugent score were assessed using a 100× oil immersion microscope. The wet tablets for AV score were examined using a low-power microscope. All slides were cross-evaluated by trained technicians.

Grading criteria for morphological indicators were established as follows: (i) Bacterial density: Recorded as grades I-IV based on the average number of bacteria per field of view (I: 1–9 bacteria/field; II: 10–99 bacteria/field; III: over 100 bacteria/field; IV: bacterial aggregation forming clusters or densely covering mucosal epithelial cells) (31). (ii) Flora diversity: Classified into grades I-IV according to the number of visible bacterial species (I: 1–3 species; II: 4–6 species; III: 7–9 species; IV: more than 10 species) (31). (iii) Predominant microbiota: Determined as the bacteria with the largest biomass or population density in the flora (29). (iv) Nugent score: Calculated based on Lactobacillus morphotypes, Gardnerella, and Bacteroides spp. Morphotypes, and Curved Gram-Variable rods (A Nugent score of 0 to 3 is considered normal; a score of 4 to 6 indicates intermediate BV; a score of ≥ 7 is diagnosed as BV) (24). (v) VVC: Diagnosed in the presence of budding yeast or hyphal forms. (vi) Trichomonas: Validated as positive when trichomonads were observed (23). (vii) AV score: Scored from 0 to 10 based on lactobacillary grades, number of leukocytes, proportion of toxic leukocytes, background flora, and proportion of polymorphonuclear leukocytes (A score of < 3 corresponds to “no signs of AV”; 3 - 4 to “light AV”; 5 - 6 to “moderate AV”; any score > 6 to “severe AV”) (25).

#### Functional examination

The measurement of five main indicators was conducted using the AV/BV combined detection kit (Beijing Zhong Sheng Jin Yu Diagnostic Technology Co., Ltd) following the manufacturer’s protocol. In brief, the swab was placed into the sample tube, and 8–10 drops of sample diluent were added. The cotton swab was then compressed to facilitate sample overflow. Using a straw, the sample liquid was extracted and dispensed into five designated holes on the test card. Subsequently, one drop of the corresponding chromogenic liquid was added to each reaction hole, and the entire setup was incubated at 37°C for 10 minutes. The reaction in each hole was observed and compared with the colorimetric card for assessment.

#### Diagnostic criteria

BV is defined by a Nugent score of ≥ 7. AV is characterized by an AV score of ≥ 3. VVC is identified by the presence of visible budding yeast or hyphal forms. Trichomonas Vaginitis (TV) is recognized by the presence of visible trichomonads. The normal vaginal microbiome (NVM) is represented by the following criteria: Bacterial density II and III, Flora diversity II and III, Presence of large gram-positive rods, Nugent score of 1 - 3, AV score < 3, and Negative H_2_O_2_ and functional indicators. The abnormal vaginal microbiome (AVM) is represented by the following criteria: Bacterial density I and IV, Flora diversity I and IV, Presence of Gram-positive cocci, large Gram-negative rods, or small Gram-negative rods, Nugent score of ≥ 7, AV score of ≥ 3, Positive H_2_O_2_ and functional indicators (**Table 1**).

**Table 1:** Vaginal microecology evaluation system.

Items	Normal	Abnormal
Morphological indicators		
Bacterial density	Grades II/III	Grades I/IV
Flora diversity	Grades II/III	Grades I/IV
Predominant microbiota	Large Gram-positive rods	Gram-positive cocci Large Gram-negative rods Small Gram-negative rods
Nugent score	1-3	≥4
AV score	<3	≥3
Pathogen	Negative	Fungus (budding yeast or hyphal forms) and/or trichomonas
Functional indicators		
H_2_O_2_	Positive	Negative
Enzymes	Negative	Positive

### Statistical analysis

The data were analyzed using IBM SPSS 25.0 statistical software. Quantitative data were represented as either mean ± standard deviation (Mean ± SD) or median. Categorical data were presented as numbers and percentages. The *t-test* was employed to analyze continuous data. The Mann-Whitney *U*-test was used to analyze non-normally distributed data. Pearson χ2 or Fisher’s exact test was used for the comparison of proportions between groups as appropriate.

Multivariate logistic regression was performed to identify factors predicting LBR. The candidate predictors of live birth were VM post-COS, VM pre-COS, age, antral follicle count (AFC), BMI, type of infertility, duration of infertility, duration of stimulation, total dosage of Gn used, Estradiol/Progesterone (E_2/_P) levels on the day of hCG administration, number of oocytes retrieved, and number of embryos transferred in the fresh cycle. Multivariate stepwise logistic regression analysis with forward selection with a significance value (P-value) set at 0.05 for addition to the model was performed with the live birth as the dependent variable and VM as the main independent variable.

## Result

### VMES is able to predict ART clinical outcomes in BV patients

**Figure 2:**
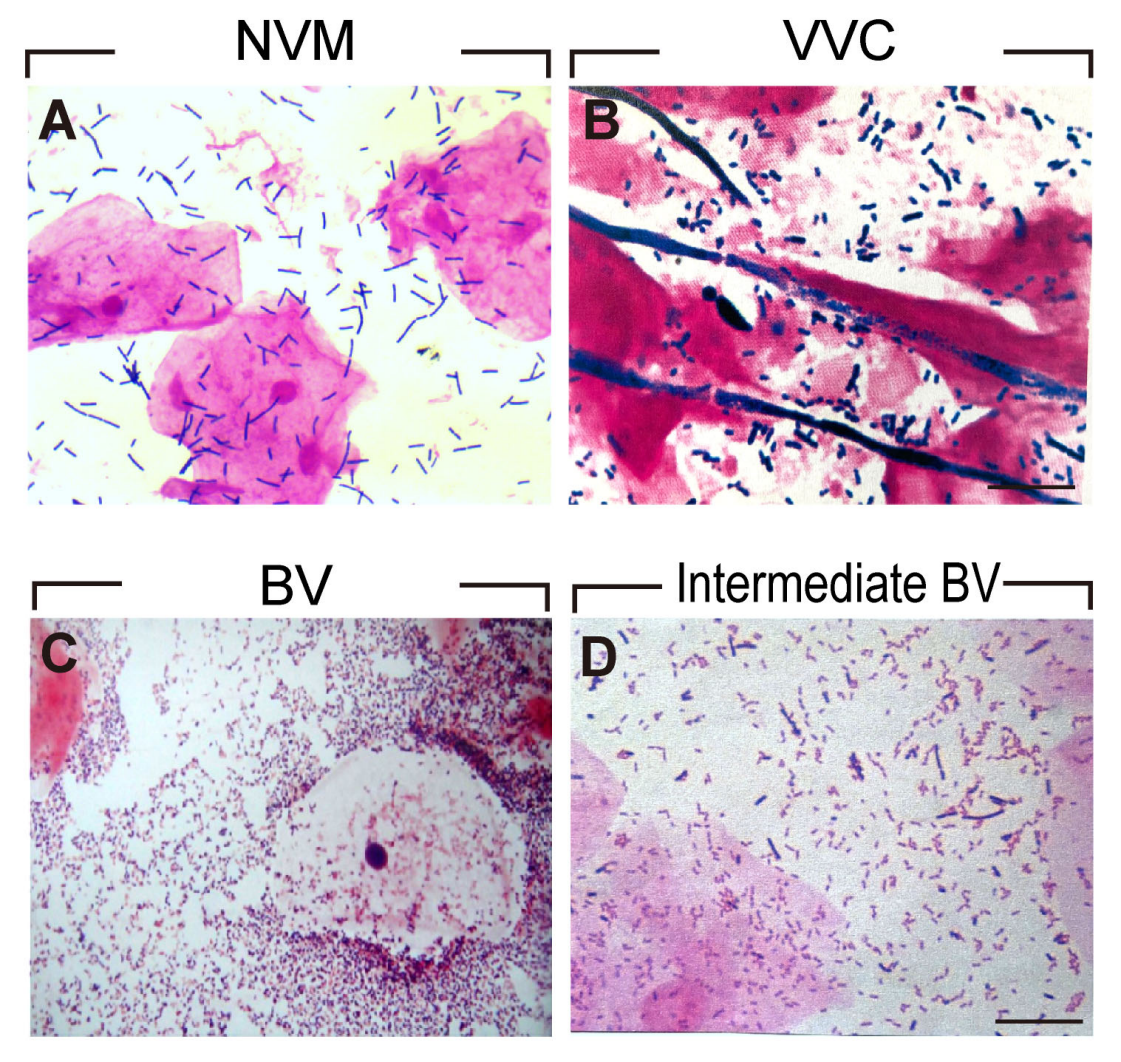
Morphological analyses of Gram-stained vaginal smears for **(A)** NVM **(B)** VVC **(C)** BV **(D)** Intermediate BV patients.

**Figure 3:**
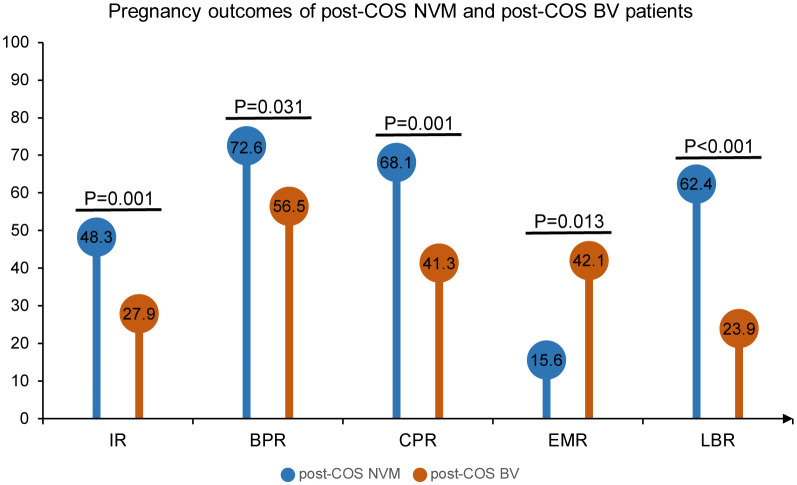
Pregnancy outcomes of post-COS NVM and post-COS BV patients. Pregnancy outcomes include IR, BPR, CPR, EMR and LBR.

To ensure the accuracy of VMES, we initially conducted tests comparing clinical outcomes between normal individuals and those diagnosed with BV. Morphological analyses of Gram-stained vaginal smears enabled the distinction between different vaginal microbiota profiles. Normal vaginal epithelial cells with distinct Lactobacillus morphotypes represented the NVM (**Figure 2A**). In the case of VVC patients, blastospores with hyphae morphotypes were visible under the microscope (**Figure 2B**). BV was diagnosed when clue cells with Gardnerella vaginalis morphotypes were observed in the smear (**Figure 2C**), while intermediate BV was determined with a Nugent score ranging from 4 to 6 (**Figure 2D**). Subsequently, we analyzed the clinical outcomes among these groups. As shown in **Figure 3**, pregnancy outcomes, including IR, BPR, CPR, and LBR, were significantly lower in post-COS BV patients compared to post-COS NVM patients (IR: 27.9% vs. 48.3%, OR = 0.41, 95% CI [0.25, 0.69], p = 0.001; BPR: 56.5% vs. 72.6%, OR = 0.49, 95% CI [0.26, 0.49], p = 0.031; CPR: 41.3% vs. 68.1%, OR=0.33, 95% CI [0.17, 0.63], p = 0.001; LBR: 23.9% vs. 62.4%, OR=0.19, 95% CI [0.09, 0.39], p < 0.001). Meanwhile, EMR was significantly higher in the post-COS BV group compared to the post-COS NVM group (42.1% vs. 15.6%, OR=3.94, 95% CI [1.44, 10.8], p = 0.013). These findings align closely with recent investigations employing the 16S rRNA gene sequencing approach (32), demonstrating that VMES is equally accurate with 16S rRNA gene sequencing in predicting ART outcomes.

### The VM undergoes dynamic changes during the ovarian stimulation process

To assess the extent to which estrogen changes influence the shift in vaginal microbiota during the ovarian stimulation process, we evaluated the microecological status of all patients before and after ovarian stimulation. The detailed prevalence of NVM and AVM in both pre-COS and post-COS samples is presented in **Table 2**. As expected, NVM was the most prevalent genus both before (pre-COS) and after (post-COS) ovarian stimulation. However, the abundance of NVM decreased in the post-COS vaginal swabs compared to pre-COS (51.4% vs. 68.0%, p < 0.001), with 16.6% (73 patients) exhibiting a shift in vaginal microbiota toward AVM across specimens. Specifically, the VVC group didn’t show a significant difference pre-COS and post-COS (3.86% vs. 4.09%, p = 0.863). However, patients diagnosed with BV and Unidentified Dysbiosis exhibited a significant increase after COS (5.91% vs. 10.5%, p = 0.014; 22.3% vs. 34.1%, p < 0.001, respectively). Among them, the pre-COS vs. post-COS difference was particularly pronounced in patients with intermediate BV and abnormal density/diversity, while no significant difference was observed in patients with abnormal predominant microbiota. Taken together, these results indicated that the VM undergoes a noticeable shift after ovarian stimulation in a substantial percentage of patients.

**Table 2:** Vaginal microecology between pre-COS and post-COS patients.

Items	Pre-COS (%)	Post-COS (%)	*χ^2^*	*P*
NVM	299 (68.0%)	226 (51.4%)	25.2	0.000
AVM	141(32.1%)	214(48.6%)	25.2	0.000
VVC	17(3.86%)	18(4.09%)	0.03	0.863
BV	26(5.91%)	46 (10.5%)	6.05	0.014
Unidentified dysbiosis ^a^	98(22.3%)	150(34.1%)	15.2	0.000
Intermediate BV	37 (8.41%)	53(12.1%)	3.17	0.075
Abnormal predominant microbiota	14 (3.18%)	14 (3.18%)	0.00	1.000
Abnormal density/diversity	47 (10.7%)	83(18.9%)	11.7	0.001

a. “Unidentified dysbiosis” includes intermediate BV, abnormal dominant flora and abnormal bacterial density/diversity.

COS, Controlled ovarian hyperstimulation; χ^2^, chi-square statistic; P, p-value; NVM, Normal vaginal microecology; AVM, Abnormal vaginal microecology; VVC, Vulvovaginal candidiasis; BV, Bacterial vaginosis.

### AVM negatively correlates with pregnancy outcomes

In order to examine the correlation between AVM and pregnancy outcomes on a larger scale, we conducted a comparative analysis using VMES between AVM and NVM patients. The study included patients undergoing the first GnRH-a prolonged protocols for IVF-ET due to tubal factors from January 2018 to December 2021. To ensure the study’s validity, various baseline characteristics were assessed, and no significant differences were found between AVM and NVM patients. These characteristics included age (33.3 ± 3.67 vs. 33.1 ± 3.78, p = 0.536), BMI (22.8 ± 2.79 vs. 22.4 ± 2.98, p = 0.172), duration of infertility (3.48 ± 2.26 vs. 3.88 ± 2.68, p = 0.097), primary infertility (54.0% vs. 58.4%, p = 0.349), basal FSH (7.26 ± 2.78 vs. 7.26 ± 2.22, p = 0.992), total dosage of Gn used (2651.9 ± 841.1 vs. 2639.0 ± 864.6, p = 0.874), duration of stimulation (11.6 ± 2.04 vs. 11.6 ± 2.28, p = 0.890), and the number of embryos transferred (1: 16.8% vs. 16.8%; 2: 83.2% vs. 83.2%; p = 0.998). These characteristics were summarized in **Table 3**.

**Table 3:** Characteristics of post-COS NVM and post-COS AVM patients.

Items	Total	post-COS NVM	post-COS AVM	t/χ2	P
Number of patients	440	226	214		
Age ( $\bar{\text{X}}$ ± s)	33.2 ± 3.72	33.3 ± 3.67	33.1 ± 3.78	0.62	0.536
BMI ( $\bar{\text{X}}$ ± s)	22.6 ± 2.88	22.8 ± 2.79	22.4 ± 2.98	1.37	0.172
Duration of infertility ( $\bar{\text{X}}$ ± s)	3.67 ± 2.48	3.48 ± 2.26	3.88 ± 2.68	-1.66	0.097
Type of infertility Primary infertility (%)	247 (56.1%)	122 (54.0%)	125 (58.4%)	0.88	0.349
basal FSH (mmol/L, $\bar{\text{X}}$ ± s)	7.26 ± 2.52	7.26 ± 2.78	7.26 ± 2.22	-0.01	0.992
Total dosage of Gn used (IU, $\bar{\text{X}}$ ± s)	2645.6 ± 851.6	2651.9 ± 841.1	2639.0 ± 864.6	0.16	0.874
Duration of stimulation (d, $\bar{\text{X}}$ ± s)	11.6 ± 2.16	11.6 ± 2.04	11.6 ± 2.28	-0.14	0.890
# Embryos transferred				0.00	0.998
1	74 (16.8%)	38 (16.8%)	36 (16.8%)		
2	366 (83.2%)	188 (83.2%)	178 (83.2%)		

COS, Controlled ovarian hyperstimulation; NVM, Normal vaginal microecology; AVM, Abnormal vaginal microecology; t, t-statistic; χ^2^, chi-square statistic; P, p-value; $\bar{\text{X}}$ , mean; s, standard deviation; IU, International Unit; d, day.

**Figure 4:**
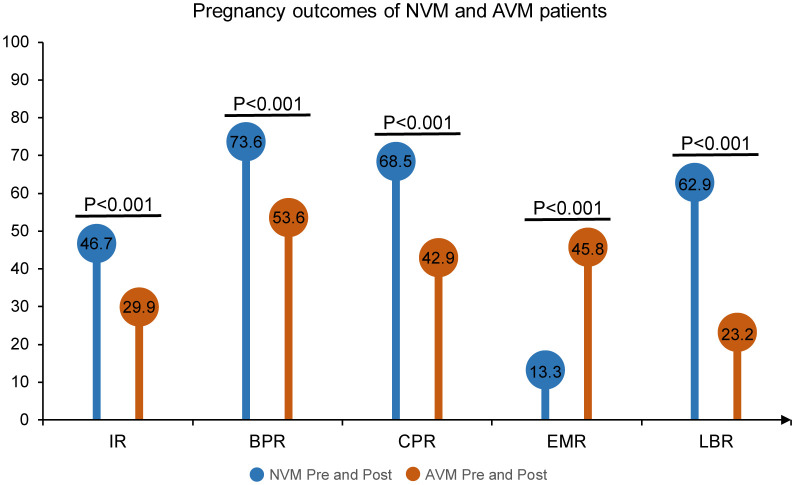
Pregnancy outcomes of NVM and AVM patients. The Pre refers to pre-COS analysis and Post to post-COS analysis. Pregnancy outcomes include IR, BPR, CPR, EMR and LBR.

In our initial comparison, we contrasted individuals identified with AVM throughout the IVF process (AVM Pre and Post) with those consistently identified with NVM throughout the process (NVM Pre and Post). The results, as presented in **Figure 4**, revealed a consistent and significant difference in pregnancy outcomes. Pregnancy outcomes, including IR, BPR, CPR, and LBR, were significantly lower in the AVM Pre and Post group compared to the NVM Pre and Post group (IR: 29.9% vs. 46.7%, OR = 0.49, 95% CI [0.34, 0.70], p < 0.001; BPR: 53.6% vs. 73.6%, OR = 0.41, 95% CI [0.25, 0.67], p < 0.001; CPR: 42.9% vs. 68.5%, OR=0.34, 95% CI [0.21, 0.56], p < 0.001; LBR: 23.2% vs. 62.9%, OR=0.18, 95% CI [0.11, 0.30], p < 0.001). Conversely, the EMR was much higher in AVM patients than in NVM patients (45.8% vs. 13.3%, OR = 5.50, 95% CI [2.59, 11.69], p < 0.001). These results further affirmed the consistent and adverse impact of AVM on clinical outcomes in IVF patients, with AVM Pre and Post patients exhibiting the highest miscarriage rate.

**Figure 5:**
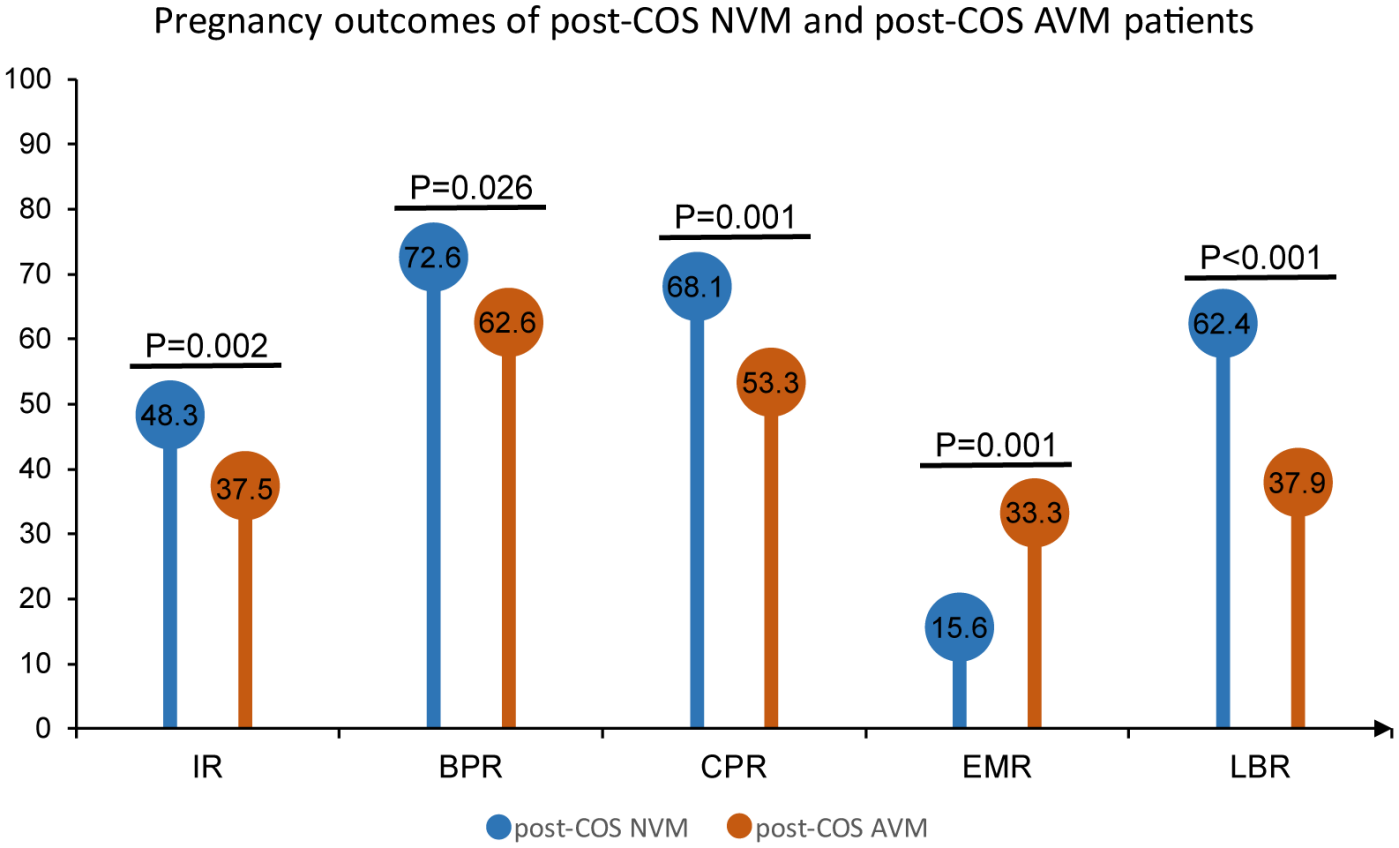
Pregnancy outcomes of post-COS NVM and post-COS AVM patients. Pregnancy outcomes include IR, BPR, CPR, EMR and LBR.

In a further examination focusing on easily identifiable predictors for pregnancy outcomes, we specifically investigated the clinical outcomes of post-COS NVM and post-COS AVM individuals. The results, outlined in **Figure 5**, highlighted that post-COS AVM patients exhibit notably poorer clinical outcomes across various categories compared to NVM patients. Specifically, IR, BPR, CPR, and LBR, were significantly lower in AVM patients compared to NVM patients (IR: 37.5% vs. 48.3%, OR = 0.64, 95% CI [0.49, 0.85], p = 0.002; BPR: 62.6% vs. 72.6%, OR = 0.63, 95% CI [0.42, 0.95], p = 0.026; CPR: 53.3% vs. 68.1%, OR=0.53, 95% CI [0.36, 0.79], p = 0.001; LBR: 37.9% vs. 62.4%, OR=0.37, 95% CI [0.25, 0.54], p < 0.001). Conversely, the EMR was much higher in AVM patients than in NVM patients (33.3% vs. 15.6%, OR=2.71, 95% CI [1.51, 4.86], p = 0.001). These findings strongly suggested that post-COS VM may serve as a predictive indicator for pregnancy outcomes.

### VM status serves as an independent indicator of pregnancy outcomes

To evaluate the association between VM and ART clinical outcomes, a multivariate stepwise regression analysis was conducted. As shown in **Table 4**, variables that exhibited a tendency of association in the univariate analysis were considered for inclusion in the multivariate stepwise logistic regression analysis (p < 0.25). Intriguingly, multivariate stepwise logistic regression analysis revealed, that both VM pre-COS and post-COS remained independent predictive factors for LBR (OR = 0.42, 95% CI [0.26, 0.673], p < 0.001; OR = 0.46 [0.29, 0.72], p = 0.001, respectively). This indicated that VM, both before and after COS, independently predicts the likelihood of achieving a live birth. In line with numerous established findings, age and the number of embryos transferred were identified as significant factors affecting LBR (OR = 0.90, 95% CI [0.85, 0.96], p = 0.001; OR = 2.61 [1.46, 4.66], p = 0.001, respectively). This suggested that both age and the number of embryos transferred are independent factors influencing the likelihood of live birth. Indeed, the findings strongly suggested that VM status, both before and after COS, served as an independent indicator for pregnancy outcomes, even when accounting for age and the number of embryos transferred.

**Table 4:** VM status serves as an independent indicator for pregnancy outcomes.

Variables	Univariate analysis			Multivariate analysis		
	B	*P*	OR (95%CI)	B	*P*	OR (95%CI)
Age	-0.12	0.000	0.89(0.84, 0.94)	-0.09	0.001	0.90(0.85, 0.96)
Type of infertility	-0.12	0.551	0.89(0.61, 1.30)			
Duration of infertility	-0.12	0.003	0.88(0.82, 0.96)	-0.08	0.083	0.92(0.84, 1.01)
BMI	-0.05	0.129	0.95(0.89, 1.02)	-0.02	0.697	0.99(0.91, 1.06)
AFC	-0.01	0.317	0.99(0.96, 1.01)			
basal FSH	-0.05	0.226	0.95(0.88, 1.03)			
basal LH	-0.01	0.673	0.99(0.95, 1.04)			
E_2_ level on the day of HCG administration	0.00	0.123	1.00(1.00, 1.00)	0.00	0.792	1.00(1.00, 1.00)
P level on the day of HCG administration	-0.09	0.315	0.92(0.77, 1.09)			
Duration of stimulation	0.06	0.165	1.06(0.98, 1.16)			
Total dosage of Gn used	0.00	0.909	1.00(1.00, 1.00)			
Number of oocytes retrieved	0.05	0.071	1.05(0.99, 1.11)	0.03	0.352	1.03(0.97, 1.11)
Number of embryos transferred	1.03	0.000	2.80(1.63, 4.80)	0.96	0.001	2.61(1.46, 4.66)
VM pre-COS	-1.22	0.000	0.30(0.19, 0.45)	-0.88	0.000	0.42(0.26, 0.67)
VM post-COS	-1.02	0.000	0.36(0.25, 0.53)	-0.78	0.001	0.46(0.29, 0.72)

VM, Vaginal microecology; B, Beta; P, p-value; OR, Odds-ratio; 95% CI, Confidence interval at 95%; BMI, Body mass index; AFC, Antral follicular count; FSH, Follicle-stimulating hormone; LH, Luteinizing hormone; E_2_, Estradiol; HCG, Human chorionic gonadotropin; Gn, Gonadotropin; COS, Controlled ovarian hyperstimulation.

## Discussion

Over the past century, the study of the microbiota in the female reproductive tract has evolved from direct cultivation methods to more sophisticated molecular techniques, particularly the sequencing of the bacterial 16S rRNA gene, which has become a pivotal approach in this domain (33). However, high-throughput sequencing technology is time-consuming, technically challenging and costly. Additionally, challenges arise from potential contamination in extraction kits and laboratory reagents, especially in samples with low microbial biomass (34). Despite advancements, challenges persist in accurately characterizing microbial diversity in the female genital tract. The transcervical approach to endometrial sample collection poses risks of contamination, necessitating cautious interpretation of microbial composition results from various reproductive tract sites. Notably, non-invasive, and reliable self-sampling approaches have primarily focused on assessing the VM, acknowledging its complexity, encompassing over 200 bacterial species in reproductive-age women (35).

Hormonal stimulation during IVF treatment induces shifts in the VM, with a pronounced increase in microbial instability in the uterine cavity, particularly after COS (8). The VMES has emerged as a valuable tool for navigating the diverse vaginal micro-ecosystem. Our study, conducted within the framework of IVF-ET, used the VMES to investigate hormonal influences on the VM. We were able to see a significant shift from NVM toward AVM among our IVF patients (**Table 2**). The prevalence of vaginal dysbiosis (VD) in infertile patients undergoing IVF treatment ranges from 17% to 19%, with variations attributed to study heterogeneity (5, 10, 19). BV, a common form of VD, is reported in approximately 19% of infertile women. Our study found a BV prevalence of 5.91% (**Table 2**), employing the Nugent score as a diagnostic standard, highlighting the importance of accurate diagnostic tools due to morphological similarities that may lead to misclassification (6, 24).

BV emerged as an independent risk factor for implantation failure, early miscarriage, pre-term birth, and low birth weight. Our findings align with meta-analyses linking BV to ART failure, including lower rates of clinical pregnancy and an association with early spontaneous abortion. Patients with BV exhibited higher rates of early spontaneous miscarriage compared to those with normal vaginal microbiota, consistent with previous studies (5, 19, 36).

While BV has received considerable attention, abnormal vaginal microbiota may not always align with BV, and diverse conditions can independently affect pregnancy outcomes. It was reported that the CPR and ongoing pregnancy rate decreased with the increase in Nugent score, while preclinical pregnancy loss and miscarriage were positively correlated with Nugent score. Regarding IR, CPR, and ongoing pregnancy rate, the differences were significant between the NVM and VD groups (37). Indeed, our study, employing the VMES to define VD, contributes to understanding its impact on reproductive outcomes in women undergoing IVF treatment. Specifically, our findings highlight the association between AVM and decreased LBR, reinforcing the influence of vaginal microbiota on pregnancy outcomes (**Figures 4**, **5**). Our study suggests that post-COS AVM patients had good predictive value in terms of LBR (37.9%) which is significantly lower than its counterparts (62.4%). Notably, AVM Pre and Post patients had the lowest live birth rate (23.2%), as compared to that of NVM Pre and Post (62.9%). In support of this, the latest randomized controlled trial demonstrated that intravaginal lactobacilli supplementation before embryo transfer in the frozen-thaw cycle significantly reduced the miscarriage rate (38). These insights may pave the way for personalized therapeutic interventions, including vaginal administration of antibiotics, prebiotics, or probiotics, with the aim of modulating AVM toward a more normal profile (39, 40). It is important to note that some recent studies have highlighted the predictive potential of the VM, in addition to known factors like duration of subfertility, a woman’s age, and body mass index (BMI), for IVF or IVF-ICSI outcomes before treatment initiation (41, 42). This study also proposes that VMES can be a valuable tool in predicting outcomes of assisted reproductive treatments, providing insights into the influence of the VM on fertility. The findings open avenues for enhancing the precision of IVF protocols, potentially reducing the physical, emotional, and financial burdens on patients.

Our data indicate that the VM is associated with ART outcomes. However, it is still unknown whether a causal link exists. The most common vaginal species considered healthy are *Lactobacillus* spp., whereas species associated with vaginal dysbiosis typically include Gardnerella vaginalis and *Atopobium vaginae*. These species have also been shown to be part of the endometrial microbiota (43–45). It seems biologically plausible that vaginal bacteria may ascend to the endometrium, potentially leading to chronic endometritis, intrauterine infection, chorioamnionitis, and uterine contractions (46–48). In addition, it was well documented that microbiota is capable of mediating immune modulation by regulating the Th17 response and the ratio of Th1 to Th17 (49). Such a mechanism would be in agreement with the hypothesized model of microbiota regulation of immune cells by interfering with carbohydrate and fat metabolism. Dysbiosis in the VM may negatively affect the implantation process in IVF-ET patients, possibly through mechanisms involving inflammation, immune dysregulation, and altered metabolic pathways (50, 51).

Our study has certain limitations, including the absence of vaginal microbiota data on the day of fresh embryo transfer and a focus on IVF-ET patients undergoing the first GnRH-a prolonged protocol due to tubal factors. Before embryo transfer, these women undergo repeated vaginal ultrasonography, vaginal flushing, and transvaginal fornix oocyte collection, which may disrupt the integrity of the vaginal flora. Therefore, how VM dynamics change during pregnancy, and their relation to ART outcomes will be a subject of future investigations. While our single-center investigation has inherent biases, the large sample size, multivariate regression model, and adjusted marginal means enhance the reliability of our conclusions. Future studies in larger multicenter prospective settings will be crucial to validating our findings.

## Data availability statement

The original contributions presented in the study are included in the article/supplementary material. Further inquiries can be directed to the corresponding authors.

## Ethics statement

The studies involving humans were approved by The Ethical Committee of the Affiliated Hospital of Qingdao University, China (Ref: QYFYWZLL26140). The studies were conducted in accordance with the local legislation and institutional requirements. The participants provided their written informed consent to participate in this study. Written informed consent was obtained from the individual(s) for the publication of any potentially identifiable images or data included in this article.

## Author contributions

QT: Data curation, Writing – original draft. SJ: Data curation, Visualization, Writing – original draft. GZ: Methodology, Writing – original draft. YjL: Data curation, Writing – original draft. JxL: Data curation, Software, Writing – original draft. XT: Resources, Visualization, Writing – original draft. YL: Software, Supervision, Writing – original draft. JL: Conceptualization, Validation, Writing – review & editing. YfiL: Conceptualization, Resources, Supervision, Writing – review & editing. ZW: Conceptualization, Funding acquisition, Writing – review & editing.
